# Unlocking the molecular secrets of *Paeonia* plants: advances in key gene mining and molecular breeding technology

**DOI:** 10.1093/hr/uhaf090

**Published:** 2025-04-30

**Authors:** Daqiu Zhao, Honglei An, Jun Tao

**Affiliations:** College of Horticulture and Landscape Architecture, Yangzhou University, Yangzhou, Jiangsu 225009, China; College of Horticulture and Landscape Architecture, Yangzhou University, Yangzhou, Jiangsu 225009, China; College of Horticulture and Landscape Architecture, Yangzhou University, Yangzhou, Jiangsu 225009, China

## Abstract

*Paeonia* plants are famous for their ornamental, medicinal, and oil values. Due to the popularity of seed oil and cut flowers in the market, the mechanisms underlying related traits of *Paeonia* plants have been fascinating, and the research work on them has increased rapidly in recent years, urging a comprehensive review of their research progress. To unlock the molecular secrets of *Paeonia* plants, we first summarize the latest advances in their genome research. More importantly, we emphasize the key genes involved in plant growth and development processes, such as bud dormancy, flowering regulation, seed oil formation, flower coloration, stem strength regulation, fragrance emission, as well as plant resistance to stress, including drought, high-temperature, low-temperature, salt, and waterlogging stresses, and biotic stress. In addition, the advances in molecular breeding technology of *Paeonia* plants are highlighted, such as molecular marker, genetic map, localization of quantitative trait loci, tissue culture, and genetic transformation system. This review covers advances in the past decades and provides valuable insights into the perspectives for the key gene mining and molecular breeding technology of *Paeonia* plants, which would help breed new *Paeonia* varieties through molecular breeding technology.

## Introduction

*Paeonia* species are highly precious ornamental plants, classified into tree and herbaceous types according to their growth morphology. Tree peony, known as the ‘King of flowers’, is famous for its gorgeous flowers that has been cultivated in China for more than 1600 years [1]. Its colors, shapes, and fragrances are diverse, making it the national flower of the Tang, Ming, and Qing dynasties. In addition to ornamental value, it also has both medicinal and oil properties. The Chinese medicine ‘Mudanpi’ is derived from the root of *Paeonia suffruticosa*, containing unique therapeutic components: paeoniflorin and paeoniflorinol [2]. The seed oil of *Paeonia ostii* is rich in unsaturated fatty acids (UFAs), fat-soluble vitamin E, and squalene, and has become a newly healthy edible oil [3]. Moreover, herbaceous peony, known as ‘Prime minister of flowers’, is also prized for its ornamental and medicinal values. In recent years, herbaceous peony has become an emerging high-end cut flower in the international market and is welcomed by consumers [4].

With the advancement of molecular biology technology, researchers have become increasingly intrigued by the mechanisms underlying trait formation in *Paeonia* plants, and the molecular regulatory networks of key horticultural traits have been gradually revealed. In this context, a preliminary genetic transformation system has been successfully established in *P. ostii* using cotyledonary nodes, resulting in the cultivation of the first in vitro regeneration plants carrying exogenous genes [5]. In the future, it is anticipated that these identified key genes will become important resources for breeding transgenic plants. Furthermore, advancements in the development of numerous molecular markers, coupled with the refinement of genetic maps and a deeper understanding of the correlation between superior traits and genotypes, have initially formed the foundation of molecular marker-assisted breeding technology. These studies not only deepen our understanding of biology, but also closely connect with the production practice, providing theoretical and technical support for molecular breeding in *Paeonia* plants. Currently, molecular breeding technology has successfully applied to various ornamental plants, including *Rosa hybrida*, *Chrysanthemum morifolium*, *Petunia hybrida*, and *Dianthus caryophyllus*, which significantly improved their market competitiveness [6, 7]. These achievements reinforce the belief that cultivating new *Paeonia* varieties through molecular breeding technology to meet market demands is no longer out of reach, but is gradually becoming a reality.

This review systematically integrates the latest molecular advancements in *Paeonia* plants research, focusing on three key aspects: genomic breakthroughs, key gene mining, and molecular breeding technologies. Besides comparing the sequencing, assembly, and utilization of existing tree peony genome data, we also emphasize the urgent need for genome sequencing of herbaceous peony based on transcriptome studies. Additionally, we categorize and discuss the key genes regulating processes such as bud dormancy, flowering regulation, seed oil formation, flower coloration, stem strength regulation, fragrance emission, and stress response. Furthermore, we highlight the advancements in molecular breeding technologies, specifically focusing on utilizing molecular markers, developing genetic maps, identifying quantitative trait loci (QTLs), and addressing the challenges associated with establishing stable genetic transformation systems. By summarizing both the achievements and limitations of current molecular research in *Paeonia* plants, this review aims to provide scientific foundation and reference for using modern technology to breed new varieties in the future.

### Genome of *Paeonia* plants

With the advent of the genomics era, *Paeonia* plants seem to lag behind in the wave of genetic research due to their complicated genome. As early as 2012, *Prunus mume* became the first ornamental plant to complete the whole genome sequencing [8, 9]. However, it was not until 2017 that a breakthrough was made in *Paeonia* plants with the publication of the first genome draft of *P. suffruticosa* ‘Luoshen Xiaochun’. The genome size was about 13.79 Gb, which was the largest among dicotyledonous plants at that time. By integrating this genome with transcriptome data, the *MADS-box* family genes were identified, leading to the proposal of a BC model that elucidated the development of petal and stamen. While this draft could serve as a starting point for exploring genetic mechanisms in *P. suffruticosa*, the assembly remained incomplete. Poor assembly quality was likely due to PacBio subreads (N50 = 14.5 kb, mean = 9.3 kb) not spanning repetitive regions and insufficient PacBio data (error-corrected data for assembly was ~20×) [10].

To overcome above limitations and achieve a better assembly, it is necessary to acquire more and longer sequencing reads. In 2022, a significantly optimized genome of *P. ostii* was constructed, achieving a genome size of 12.28 Gb with high contiguity (contig N50 = 228 kb, scaffold N50 = 2.43 Mb). It also succeeded in anchoring 93.5% of the sequence to five chromosomes with minimal allelic overlap. Furthermore, Benchmarking Universal Single-Copy Orthologs (BUSCO) evaluation confirmed the high quality of the assembly, yielding 94.4% orthologous gene set representation, which was consistent with the high mapping coverage of the transcriptome. Many plants possessed giga-genomes; however, those consisting of giga-chromosomes like *P. ostii* (1.78–2.56 Gb) were relatively uncommon. And the rapid expansion of long terminal repeat sequences in intergenic regions over a brief evolutionary timescale (~ two million years) might contribute to *P. ostii*’s giga-genome. Meanwhile, the expansion of five histone encoding genes was also crucial for maintaining the integrity of its giga-chromosomes. Additionally, compared to *P. ostii*, the ectopic expression of *PsAP1* and the down-regulation of *PsAG* were found to be the cause of the stamen petalisation in *P. suffruticosa*, which increased the number of petals and enriched the diversity of flower types [11]. This finding provided further insight into the mechanism of unique floral organ regulation in tree peony.

Based on these two tree peony reference genomes, heterozygous single nucleotide polymorphisms (SNPs) and Sanger sequencing were used to trace the parental composition of *Paeonia* Itoh hybrid. It was found that the maternal herbaceous peony provided only one gene copy, while the paternal tree peony contributed two heterozygotic copies for Itoh hybrids. These genome-based findings revealed the molecular formation of the parental contribution in the *Paeonia* Itoh hybrid and could facilitate breeding new triploid varieties [12].

Obtaining more tree peony genomes can greatly improve our understanding of the evolutionary history of this species and accelerate the development of genome-assisted breeding techniques. Among them, *Paeonia ludlowii*, a rare pure yellow wild tree peony that grows on the Tibetan Plateau, is extremely precious for its unique genetic background and breeding value. Its genome assembly size (10.6 Gb) was slightly smaller than *P. suffruticosa* (13.79 Gb) and *P. ostii* (12.28 Gb), but *P. ludlowii* exhibited a significant quality advantage with an N50 value of 1.15 Mb, which was 23 or 5 times as much as that of *P. suffruticosa* or *P. ostii*, respectively. Further studies revealed that this genome had significant sequence differences compared to other tree peony genomes, including about 75% of specific sequence and gene level differentials, which might help it to adapt to the cold plateau environment. Comparative genomic analysis revealed that chromosome rearrangements and the centromere played a role in the evolution of *P. ludlowii* giga-chromosomes. In addition, bursts of transposable element and DNA methylation had an impact on genome size expansion and gene duplication, as well as on oil biosynthesis and flower traits [13].

These tree peony genomic resources have greatly accelerated its genetic breeding research (Table 1). Regarding herbaceous peony, although some achievements have been made at the level of chloroplast and mitochondrial genomes, which have laid a foundation for mining genetic resources and illustrating phylogenetic relationships [14, 15], its whole genome has not yet been completed. Therefore, it is crucial to bridge the gap in herbaceous peony genome research. At present, abundant transcriptome research has been conducted on various herbaceous peony varieties. Transcriptome sequencing not only reveals gene expression patterns under different conditions and aids in gene annotation and function prediction, but also improves the completeness and accuracy of genome assembly through high-quality data. In addition, these studies provide molecular clues to resolve gene regulatory mechanisms (e.g., floral development and environmental adaptation) and drive innovation in genomic analysis methods (e.g., comparative transcriptome and evolutionary studies). In the absence of a complete genome, transcriptome data have become an important cornerstone for genomic studies of herbaceous peony, laying the foundation for further construction of their own genome.

**Table 1:** Reference genome information of *Paeonia* plants.

**Code**	**Date**	**Accession number**	**Species**	**Method**	**Genome size**	**N50 length**	**Completeness score**	**Predicted genes**	**Genes matched known proteins**	**Features**	**Reference**
1	24 December 2018	CNP0000281	*P. suffruticosa* ‘Luoshen Xiaochun’	*De novo* sequencing and assembly	13.78 Gb	49.94 Kb	61.2%	35 687	89.35%	The first draft genome of *Paeonia* plants; less complete assembly.	[10]
2	3 October 2022	CNP0003098	*P. ostii* ‘Feng Dan’	*De novo* sequencing and assembly	12.28 Gb	228 Kb	94.4%	73 177	84.53%	Chromosome-level genome assembly; high-continuity assembly; high percentage of chromosomal anchoring.	[11]
3	18 November 2023	PRJCA016714	*P. ludlowii*	*De novo* sequencing and assembly	10.33 Gb	1.15 Mb	98.5%	46 582	99.35%	The highest quality assembly; genome from wild tree peony; large sequence and structural variations compare to other *Paeonia* genomes.	[13]
4	5 November 2024	PRJCA026015	*Paeonia* Itoh hybrid ‘Bartzella’	Whole-genome resequencing	–	–	–	–	–	Resolving hybrid parental contributions based on SNPs and Sanger sequencing.	[12]

## Key genes regulating plant growth and development

### Bud dormancy

**Figure 1:**
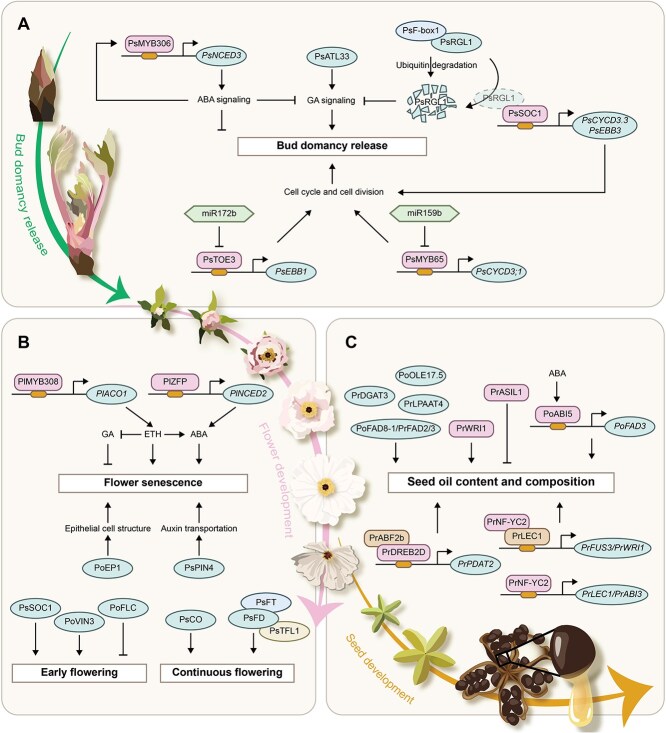
Key genes regulating plant growth and development in *Paeonia* plants. (A) Regulatory network related to bud dormancy. (B) Regulatory network related to flowering events. (C) Regulatory network related to seed oil formation. ABA: abscisic acid; ETH: ethylene; GA: gibberellin.

High winter temperatures in southern China lead to insufficient accumulation of cold required for bud dormancy release, which severely hinders the southward plantation of *Paeonia* plants. Studies on dormancy, chilling requirement (CR), and related molecular mechanisms in *Paeonia* plants are needed to expand the cultivation range (Fig. 1A).

Carbohydrates are a source of energy for plant growth and development. Starch degradation and Embden–Meyerhof–Parnas pathway (glucose metabolic pathway) activation provided energy and material basis for flavonoid accumulation during *P. suffruticosa* endodormancy release [16]. By further comparing two *Paeonia lactiflora* cultivars with contrasting CRs, it was concluded that ‘Zhuguang’ (high CR) had lower expression of genes related to starch metabolism and sucrose biosynthesis, which was consistent with its lower content of soluble sugars and ultimately made it difficult to break dormancy due to lack of energy supply [17]. When transcriptome sequencing was performed using *P. lactiflora* ‘Hang Baishao’ buds, *PlSOC1* and *PlWRKY33* were found to be crucial in determining its low CR characteristics [18]. This might facilitate the breeding of new low CR varieties for horticultural applications in the subtropics.

Endogenous hormones, particularly gibberellin (GA) and abscisic acid (ABA), play crucial roles in regulating bud endodormancy. Their relationship is antagonistic: high ABA expression occurs in dormant buds, while high GA levels aid in breaking dormancy. Transcriptomic data showed that genes involved in GA biosynthesis, signaling, and response play a central role in endodormancy release of *P. suffruticosa* [19]. For example, *PsATL33* positively regulated bud dormancy release by modulating GA production [20]. On the other hand, DELLA proteins are key components of the GA signaling pathway, negatively regulating GA signaling and hindering plant growth and development. The ubiquitination-dependent degradation of the DELLA protein PsRGL1 was facilitated by PsF-box1, a component of the SCF E3 ubiquitin ligase complex, ultimately leading to the release of bud dormancy [21]. PsRGL1 was also found to interact with PsSOC1, which activated the cell cycle by directly binding to the CArG motifs of the *PsCYCD3.3* and *PsEBB3* promoters. The interaction with PsRGL1 inhibited the DNA-binding activity of PsSOC1 until this inhibition was relieved by the degradation of PsRGL1, which in turn accelerated bud dormancy release [22]. In contrast to GA, ABA usually maintains bud endodormancy and acts as an inhibitor in the process of plant dormancy release. A previous study showed that PsMYB306 targeted *PsNCED3* to increase ABA production, and ABA repressed the transcription of GA pathway genes, further inhibiting bud dormancy release [23]. Interestingly, calcium might partially regulate bud dormancy release through GA and ABA pathways [24].

Cell division was gradually reinitiated and accelerated at the end of endodormancy in *P. suffruticosa* [25]. MicroRNAs were involved in this kind of cell cycle regulation. In response to chilling treatment, miR172b was downregulated while its target gene, *PsTOE3*, was induced. Furthermore, PsTOE3 could directly bind to the promoter of *PsEBB1*, which activated *PsEBB1* expression and indirectly accelerated the cell cycle via *PsCYCD*, ultimately leading to bud dormancy release and flowering [26]. A similar miR159b-PsMYB65 module could directly target *PsCYCD3;1*, which helped resume bud growth after natural dormancy. Specifically, the increase in endogenous GA in this module facilitated the expression of *PsMYB65*, which further promoted cell cycle and cell division [27].

### Flowering regulation

The short flowering time of *Paeonia* plants, usually less than a week, has become a key limitation to their ornamental value under natural conditions. Therefore, slowing down the senescence of flowers is an important objective in *Paeonia* plants cultivation (Fig. 1B). A study on flower senescence in *P. ostii* found that *PoEP1* shortened the flowering time by affecting epithelial cell structure and function [28]. Moreover, phytohormones play crucial roles in regulating plant growth, development, and senescence. Exogenous application of indole-3-acetic acid and 2,3,5-triiodobenzoic acid could extend or shorten the flowering time of *P. suffruticosa*, respectively. In this process, PsPIN4 functioned as an auxin efflux transporter and significantly prolonged flowering by influencing petal abscission initiation [29]. Moreover, ABA and ethylene (ETH) treatments accelerated petal senescence of *P. lactiflora* ‘Hang Baishao’, but GA delayed this process. The PlMYB308-*PlACO1* module positively regulated ETH production and affected ABA and GA biosynthesis, thereby accelerating flower senescence [30]. Also involving the combined action of ABA, ETH, and GA, zinc-finger protein PlZFP accelerated *P. lactiflora* flower senescence by activating the ABA biosynthesis-related gene *PlNCED2* expression [31].

Moreover, the concentrated flowering period also restricted the development of the *Paeonia* plant industry. *SOC1* plays an essential role in integrating multiple flowering signals to regulate the transition from vegetative to reproductive development. *PsSOC1* was found to accelerate bud dormancy release and promote early flowering [32]. *PoVIN3* was identified as a key gene of vernalization pathway, and the flowering period of transgenic plants with *PoVIN3* was significantly earlier than that of the wild type [33]. In contrast, *PoFLC* might play a delaying role in flowering period [34]. By comparing early flowering cultivar with the late one, small RNAs were found to play key regulatory roles in flowering period, such as miR166g-5p and miR319 [35, 36]. Besides, most tree peony cultivars in gardens are once-flowering, and promoting flowers to rebloom multiple times in a year is also a way to enhance their ornamental values. *Paeonia* × *lemoinei* ‘High Noon’ stands out as one of the rare cultivars exhibiting the continuous flowering trait. PsFT (floral promoter) and PsTFL1 (floral inhibitor) both interacted with PsFD to jointly regulate the continuous flowering trait in ‘High Noon’ [37]. Additionally, *PsCO* was another crucial reflowering-promoting gene identified in ‘High Noon’ [38].

### Seed oil formation

Seed oil of *Paeonia* plants is rich in UFAs, which is important for improving dietary structure and human health (Fig. 1C). Of these, *P. ostii* and *Paeonia rockii* are the two main oil tree peony species with large-scale cultivation in recent years [3]. Moreover, it was worth noting that *P. lactiflora* was also important in oil use, with its oil content as high as 32.71% and the UFA content ranging from 94.38% to 95.09%, with oleic acid (OA), linoleic acid (LA), and alpha-linolenic acid (ALA) being the three principal UFAs. Among them, *P. lactiflora* ‘Hang Baishao’ and ‘Fenghuang Niepan’ exhibited considerable seed yields (30.75 and 25.63 g per stem, respectively) and high seed oil content (33.34% and 32.56%, respectively), qualifying them as oil cultivars [39].

Fatty acid desaturase is a key enzyme in plant lipid metabolism, which can promote the conversion of fatty acids (FAs) to UFAs. Integrated analysis of transcriptomic and proteomic data from *P. ostii* seeds revealed that 53–88 days after pollination was a critical period for oil biosynthesis and FA metabolism, and *PoFAD* helped ALA accumulation [40]. Furthermore, *PrFAD3* was found to be involved in ALA biosynthesis through the phosphatidylcholine-derived pathway because of its high expression in *P. rockii* with abundant ALA content [41]. And overexpression of *PrFAD2* and *PrFAD3* could increase LA and ALA content, respectively [42]. *PoFAD8-1* also had a close relationship to the high content of ALA in *P. ostii* seed oil [43]. Moreover, diacylglycerol acyltransferase gene (*PrDGAT3*) was important in catalyzing triacylglycerol (TAG) biosynthesis, with a substrate preference for UFAs, especially LA and ALA [44]. Overexpression of lysophosphatidic acid acyltransferase gene (*PrLPAAT4*) could enhance OA and FA contents, and also upregulated TAG assembly-related gene expression levels, suggesting that it could act as a positive regulator of seed FA biosynthesis [45]. Additionally, oil bodies (OBs) serve as the organelles responsible for lipid storage in plants. Zhao *et al.* characterized the characteristics of *P. ostii* seed OBs and found the oleosin (*PoOLE17.5*) was a key gene related to its morphology [46]*.*

In many higher plants, seed oil accumulation is governed by complex multilevel regulatory networks including transcriptional regulation. For instance, the AP2 family transcription factor PrWRI1 of *P. rockii* increased FA content in transgenic *Arabidopsis* seeds and upregulated most genes related to FA biosynthesis and TAG assembly [47]. Integration of transcriptome and proteome revealed the role of PoABI5-*PoFAD3* module in regulating UFA biosynthesis during *P. ostii* seed development [48]. The dehydration-responsive element binding transcription factor PrDREB2D, as an upstream regulator of *PrPDAT2*, enhanced ALA accumulation in seeds by recruiting the cofactor ABA-response element binding factor PrABF2b [49]. The nuclear factor Y transcription factor PrNF-YC2 directly activated *PrLEC1* and *PrABI3* alone, and indirectly activated *PrFUS3* and *PrWRI1* expression via interacting with PrLEC1, and this complex network greatly promoted seed oil accumulation [50]. Conversely, the trihelix transcription factor PrASIL1 repressed FA accumulation and altered its composition by downregulating numerous seed oil biosynthetic genes [51].

### Flower coloration

Flower color is one of the most valuable traits of ornamental plants. Pigments accumulated in the vesicles of petal epidermal cells have been verified to determine the flower color. Flavonoids are the most important pigment group and produce the widest spectrum of colors, ranging from pale yellow to blue-purple [52]. In *Paeonia delavayi*, apigenin 7-*O*-neohesperidoside and chrysoeriol 7-*O-*glucoside flavonols were the primary copigments responsible for its yellow coloration, while anthocyanins contributed to its purple-red coloration [53]. Moreover, anthocyanin concentration was significantly correlated with the intensity of flower color in *P. ostii*, which resulted in flower colors ranging from almost white and light pink to deep pink [54].

**Figure 2:**
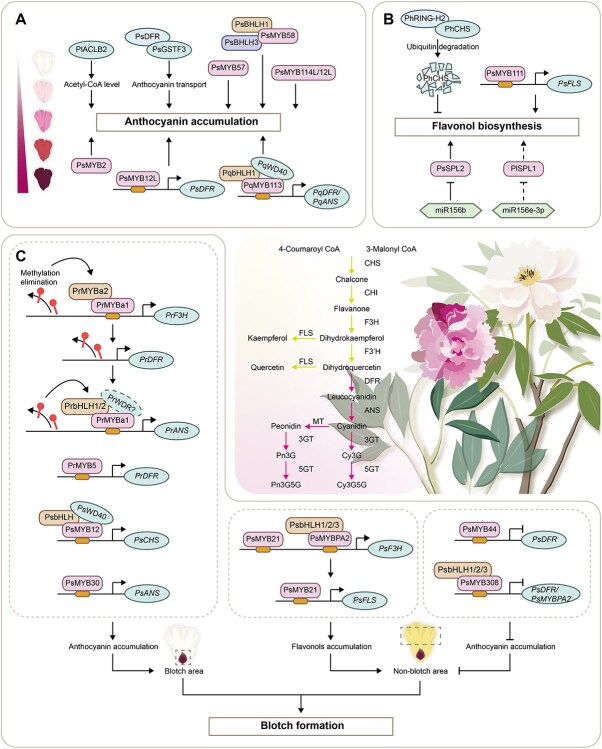
Key genes regulating flower coloration in *Paeonia* plants. (A) Regulatory network related to anthocyanin accumulation. (B) Regulatory network related to flavonol biosynthesis. (C) Regulatory network related to blotch formation. 3GT: flavonoid 3-*O*-glucosyltransferase; 5GT: flavonoid 5-*O*-glucosyltransferase; ANS: anthocyanidin synthase; CHI: chalcone isomerase; CHS: chalcone synthase; Cy3G: cyanidin-3-*O*-glucoside; Cy3G5G: cyanidin-3,5-di-*O*-glucoside; DFR: dihydroflavonol 4-reductase; F3H: flavanone 3-hydroxylase; F3′H: flavonoid 3′-hydroxylase; FLS: flavonol synthase; MT: methyltransferases; Pn3G: peonidin-3-*O*-glucoside; Pn3G5G: peonidin-3,5-di-*O*-glucoside.

In recent years, various flavonoid biosynthetic genes have been found to regulate flower color in *Paeonia* plants. In *P. lactiflora* ‘Huang Jinlun’ (yellow), low *PlCHI* expression led to chalcone accumulation, while low *PlDFR* expression prevented anthocyanin formation. In *P. lactiflora* ‘Yulou Hongxing’ (white), high expression of upstream genes combined with low *PlDFR* expression promoted abundant anthoxanthin production, accompanied by a small amount of colorless anthocyanin, while in *P. lactiflora* ‘Hongyan Zhenghui’ (red), high expressions of *PlDFR*, *PlANS*, and *PlUF3GT* converted a large number of anthoxanthins into colored anthocyanins [55]. In particular, *DFR* and *ANS* are two genes that might play key roles in anthocyanin biosynthesis, leading to the change of herbaceous peony from white to red [56, 57]. These flavonoid biosynthetic genes are often regulated by the MBW (MYB-bHLH-WD40) protein complex. For example, PqMYB113 formed a complex with PqbHLH1 and PqWD40, which activated *PqDFR* and *PqANS* expression to enhance anthocyanin accumulation in *Paeonia qiui* [58]. Similarly, PsMYB2, PsMYB57, PsMYB58, PsMYB114L, and PsMYB12L also exhibited this function [59–62]. In addition to transcriptional regulation, at the level of post-translational modification, PhRING-H2 interacted with PhCHS, thereby mediating its degradation in flower development of *Paeonia* ‘He Xie’ [63]. Except these, *PlACLB2* promoted anthocyanin accumulation by increasing the abundance of its precursor substrate acetyl-CoA level [64]. As an anthocyanin-related glutathione S-transferase, PsGSTF3 interacted with PsDFR and together contributed to the petal coloration [65] (Fig. 2A).

Pure yellow cultivars of *Paeonia* plants are rare and have a promising market; thus, this petal coloring mechanism needs to be elucidated in depth. In ‘High Noon’, flavonoid biosynthetic genes showed a unique expression pattern, with downregulated *PsC4Hs*, *PsDFRs*, and *PsUFGTs* as well as upregulated *PsFLSs*, *PsF3Hs*, and *PsF3'Hs* during initial bloom, and PsMYB111 activated *PsFLS* expression, which increased flavonol accumulation but reduced anthocyanins [66]. On the other hand, the miR156b-PsSPL2 module also contributed to the yellow petal coloration in ‘High Noon’ [67]. Similarly, the miR156e-3p-PlSPL1 module contributed to the formation of yellow inner petals in *P. lactiflora* with bicolored flowers [68]. In *P. lactiflora* ‘Huang Jinlun’, specific DNA methylation at the mC-16 locus inhibited PlC/EBPα binding to the *PlCHI* promoter, thereby regulating tissue-specific expression of the *PlCHI* [69] (Fig. 2B).

*Paeonia suffruticosa* ‘Shima Nishiki’ is a highly valued bicolored cultivar, and multilevel studies have been conducted on the molecular mechanism of bicolor petal formation. In terms of transcriptional regulation, *PsDFR* was highly expressed in its red petals, and its expression could be activated by PsMYB12L [70, 71] (Fig. 2A). Methylation might be another reason for bicolor formation, as red petals were more methylated (58.45%) than pink ones (44.36%) at the initial bloom stage, with differential expression of the methylated *PsbHLH1* in red versus pink petals [72]. Additionally, miR858 and miR156a-5p were possibly involved in regulating bicolor formation of ‘Shima Nishiki’ [73].

Petal blotch not only attracts pollinators, but also enhances the ornamental value of flowers. Different MYBs can form a complex regulatory network to control petal blotch formation. For example, the PsMYB12-PsbHLH-PsWD40 complex directly activated *PsCHS* expression, which was specific to the petal blotch in *P. suffruticosa* ‘Qinghaihu Yinbo’ [74]. The PrMYB5-*PrDFR* and PsMYB30-*PsANS* modules regulated anthocyanin accumulation at the petal base of *P. rockii* and ‘High Noon’, respectively [75, 76]. Apart from these, the mechanism of coloring inhibition in nonblotched petals has also been studied. *PsMYB44* was highly expressed in the nonblotched region of ‘High Noon’, and PsMYB44 negatively regulated anthocyanin biosynthesis by inhibiting *PsDFR* expression [77]. Synergistic actions of three MYBs underpinned blotch formation in ‘High Noon’, including PsMYB21, PsMYBPA2, and PsMYB308 [78]. Furthermore, methylation of the promoter repressed the transcription of *PrF3H*, *PrDFR*, and *PrANS* in the nonblotched region of *P. rockii*, whereas elimination of methylation in the blotched region facilitated genes activation by the PrMYBa1-PrMYBa2 and PrMYBa3-PrbHLH1/2 complex [79] (Fig. 2C).

### Stem strength regulation

**Figure 3:**
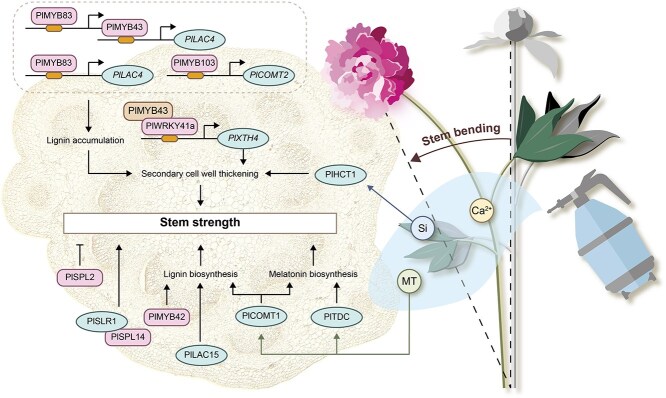
Key genes regulating stem strength development in *Paeonia* plants. Ca: calcium; MT: melatonin; Si: silicon.

As an emerging high-end cut flower, stem bending caused by insufficient stem strength is an important limiting factor for herbaceous peony. Thus, it is an urgent problem to be solved (Fig. 3). In a pervious study, stem diameter has been identified as the most intuitive indicator of *P. lactiflora* stem strength, and lignin provided mechanical support to the *P. lactiflora* stems [80]. Further study on *P. lactiflora* ‘Xixia Yingxue’ (bending) and ‘Hongfeng’ (upright) revealed that ‘Hongfeng’ exhibited significantly higher stem strength, S-lignin, G-lignin, and total lignin contents, as well as greater activities of lignin biosynthesis-related enzymes compared to ‘Xixia Yingxue’ [81]. Additionally, pectin might be another important cell wall substance affecting the stem straightness [82].

Transcription factors regulated lignin biosynthetic genes to control *P. lactiflora* stem strength. For one thing, the R2R3-MYBs (PlMYB43, PlMYB83, and PlMYB103) activated the monolignol biosynthetic gene *PlCOMT2* or the lignin polymerization-related gene *PlLAC4* expression, which enhanced *P. lactiflora* stem strength by regulating lignin biosynthesis and secondary cell wall thickening. And PlMYB83 could also act as a transcriptional activator of *PlMYB43* to further promote *PlLAC4* expression [83]. Additionally, PlMYB42 and *PlLAC15* might all enhance stem strength through promoting lignin accumulation [84, 85]. For another, XTHs were considered key enzymes in plant cell wall remodeling; the *PlXTH4* expression could be activated by the PlWRKY41a-PlMYB43 complex, functioning as a positive regulator to enhance *P. lactiflora* stem strength [86]. In addition, PlSPL14 interacted with PlSLR1 to positively regulate *P. lactiflora* stem strength [87], whereas PlSPL2 negatively regulated stem development in *P. lactiflora* [88].

Applying exogenous substances is a simple and convenient method to enhance structural carbohydrate accumulation in production, and recent studies have shown that exogenous calcium, silicon, and melatonin all could enhance *P. lactiflora* stem strength. Spraying CaCl_2_ increased endogenous calcium concentration and cell wall fractions of *P. lactiflora* stems [89]. Similarly, nano-CaCO_3_ treatment thickened the sclerenchyma cell walls and significantly enhanced lignin accumulation [90]. When ethyl glycol tetraacetic acid (EGTA, Ca^2+^ chelator) was used, it triggered the loss of Ca^2+^ from the cell wall, affected the expression of genes involved in secondary wall biosynthesis, and reduced the deposition of lignin in the xylem cells, which in turn reduced *P. lactiflora* stem strength [91]. Silicon application similarly enhanced *P. lactiflora* stem strength and differentially affected *PlHCT1* expression. Overexpression of *PlHCT1* increased secondary cell wall thickness and layers, and promoted lignin accumulation, which enhanced stem strength [92]. Application of exogenous melatonin promoted endogenous melatonin biosynthesis, which significantly improved *P. lactiflora* stem strength by increasing S-lignin content and upregulating lignin biosynthesis gene expression. Among them, both *PlTDC* and *PlCOMT1* affected melatonin biosynthesis [93].

### Fragrance emission

The fragrance of *Paeonia* flowers attracts pollinators and serves as a key indicator of their horticultural value and potential applications. In studies of tree peony floral fragrance, Li *et al.* identified 128 volatile components, predominantly terpenes, alcohols, and esters. Transcriptome analysis further revealed that, compared to ‘Feng Dan’ (faint fragrance), ‘Huang Guan’ (strong fragrance) exhibited heightened expression of genes encoding key terpenoid backbone biosynthesis enzymes (AACT, HMGR, PMK, DXS, DXR, HDS, HDR, GGPS) and monoterpene synthases (LIS, MYS), suggesting a close correlation between these genes and floral fragrance intensity [94]. In herbaceous peony, analysis of 17 varieties identified 68 volatile components, mainly terpenoids (60.94%), fatty acid derivatives (24.57%), and benzenoids/phenylpropanoids (14.49%). Among them, linalool, citronellol, geraniol, and phenylethyl alcohol (2-PE) were identified as the key aroma compounds. Further studies revealed that the different gene expressions in monoterpene and 2-PE synthesis pathways are associated with intensity variations of herbaceous peony fragrance [95].

Since *Paeonia* plants’ floral fragrance mainly comes from terpenoids, isolating and studying terpene synthesis genes is crucial. 3-Hydroxy-3-methyl-glutaryl CoA reductase (HMGR) is the first key rate-limiting enzyme in the mevalonate (MVA) pathway, which regulates the content of linalool and other floral components. This is illustrated by the increased linalool content in the *PsHMGR1* transgenic lines [96]. In the downstream stages of the terpene biosynthesis pathway, terpene synthase (TPS) is directly involved in terpene products synthesis, thereby determining their structural and functional diversity. Although there was no significant change in the linalool content of the *PsTPS1* overexpression lines, unexpectedly, the sesquiterpene volatile compound germacrene D was produced [96]. In contrast, both *PdTPS1* and *PdTPS4* of *P. delavayi* efficiently catalyzed the generation of linalool *in vitro* experiments, and overexpressing these genes *in vivo* similarly accumulated large amounts of linalool. Notably, the differential expression patterns of *PdTPS1* and *PdTPS4* might explain the abundant presence of linalool in subsect. *Delavayanae* versus its absence in subsect. *Vaginatae*, providing molecular-level insights into floral fragrance diversity and differentiation [97]. The tree peony cultivar ‘High Noon’ serves as a valuable breeding resource due to its intense fragrance, primarily attributed to linalool [98]. Transient expression of its *PsTPS14* gene in weakly scented ‘Feng Dan’ significantly enhanced linalool release, accompanied by elevated linalool synthase activity and concentration [99]. Furthermore, the spatiotemporal expression pattern of the linalool synthetase gene (*LIS*), another member of the TPS family, was highly consistent with linalool emission dynamics. Functional validation via overexpression or silencing of *PsLIS* directly altered linalool levels, reinforcing the essential role of TPS family genes in floral fragrance regulation [100]. Although floral fragrance influences consumer choice, it is often neglected in traditional breeding, leaving some *Paeonia* plants with overpowering or unpleasant scents. In the future, exploration of other aromatic components and regulatory networks will be the key to the study of floral fragrance in *Paeonia* plants.

Growth and development regulatory genes in *Paeonia* plants have delved into multiple facets, such as bud dormancy, flowering regulation, seed oil formation, flower coloration, stem strength regulation, and fragrance emission. Among them, the flower coloration mechanism, centered on the MBW complex, is the most advanced but lacks novelty due to its homogeneous research approach. In contrast, studies on bud dormancy release mechanisms are more multidimensional, encompassing ubiquitin modification, miRNA regulation, and hormonal signaling pathways (like ABA and GA), providing a comprehensive perspective for other studies to reference. In addition, the importance of stem strength trait has been emphasized in recent years. These studies have not only revealed the molecular regulatory networks but also introduced practical methods such as exogenous application of calcium, silicon, and melatonin to enhance it. While some progress has been made in identifying key regulatory genes involved in plant growth and development, our understanding of unique ornamental traits such as stamen petaloidy and spring-color foliage remains limited. Similarly, as an important nutrient storage organ, the fleshy root directly influences vegetative and reproductive growth in the subsequent year, necessitating enhanced research in this area. The unique growth patterns of *Paeonia* plants suggest specific gene regulatory networks. Discovering these networks will not only reveal their biological characteristics but also provide valuable genetic resources for molecular breeding.

## Key genes regulating plant resistance to stress

### Abiotic stress

#### Drought stress

Water deficit is a common problem *for *Paeonia** plants in central and northwestern China, which not only inhibits leaf and flower growth, but also reduces seed yield. Integrated physiological and transcriptomic analysis revealed that drought stress significantly triggered the oxidative stress response in *P. ostii*, manifested by the accumulation of reactive oxygen species (ROS) and membrane lipid peroxidation, and drought stress also damaged chloroplast structure, inhibited photosynthetic efficiency, and ultimately led to leaf wilting [101].

**Figure 4:**
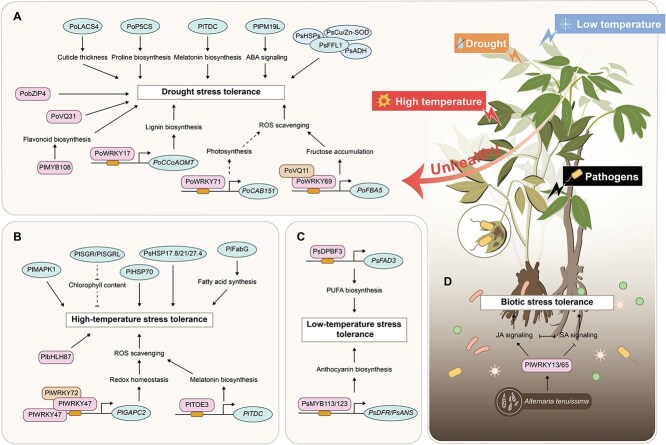
Key genes regulating *Paeonia* plants resistance to stress. (A) Regulatory network related to drought stress tolerance. (B) Regulatory network related to high-temperature stress tolerance. (C) Regulatory network related to low-temperature stress tolerance. (D) Regulatory network related to biotic stress tolerance. ABA: abscisic acid; JA: jasmonic acid; PUFA: polyunsaturated fatty acid; SA: salicylic acid.

*Paeonia* plants respond to drought stress through multiple mechanisms (Fig. 4A). *PoLACS4* could increase cuticle thickness to improve drought resistance in *P. ostii* [102]. *PoP5CS* promoted proline biosynthesis, which acted as an osmoregulator to stabilize intra- and extracellular osmotic balance [103]. *PlTDC* promoted melatonin biosynthesis and subsequently led to a reduction in the accumulation of hydrogen peroxide (H_2_O_2_) and superoxide anion radicals (O2^·−^) [104]. Meanwhile, overexpression of the plasma membrane protein *PlPM19L* increased ABA content and decreased H_2_O_2_ content, thus enhancing drought tolerance through the ABA signaling pathway [105]. In addition, a novel F-box protein PsFFL1 interacted with drought-responsive proteins (PsCu/Zn-SOD, PsADH3, and PsHSPs) and activated the expression of drought-resistant genes [106].

Transcription factors play a crucial regulatory role in response to abiotic stress. The basic leucine zipper transcription factor family member PobZIP4 positively regulated drought tolerance of *P. ostii* [107]*.* PlMYB108 significantly promoted flavonoid accumulation, enhanced the scavenging of ROS, and optimized photosynthetic efficiency to enhance drought tolerance in *P. lactiflora* [108]. Luan *et al.* explored the complex regulatory network of WRKYs in *P. ostii*, and found that PoWRKY17/69/71 all positively regulated plant drought tolerance. Firstly, PoWRKY17 could endow *P. ostii* with drought tolerance by activating the lignin biosynthetic gene *PoCCoAOMT* [109, 110]. Secondly, PoWRKY71-*PoCAB151* module stabilized photosynthesis by regulating chloroplast homeostasis and chlorophyll content under drought stress [111]. Lastly, PoWRKY69 directly bound to the W-box of the *PoFBA5* promoter to activate its expression, and PoVQ11 interacted with PoWRKY69 to strengthen the activation, which promoted fructose accumulation to enhance drought tolerance in *P. ostii* [112]. Another member of the VQ family, PoVQ31, was also a positive regulator of *P. ostii* drought tolerance by scavenging ROS [113].

#### High-temperature stress

*Paeonia* plants prefer cool weather; however, the high summer temperature in the middle and lower reaches of the Yangtze River limits their growth and development. High-temperature stress not only damaged cell membranes, increased relative conductivity (REC), malondialdehyde (MDA) content, and ROS accumulation, but also affected photosynthetic efficiency along with stomatal closure and chloroplast damage in *P. suffruticosa* [114]. Therefore, screening of cultivars adapted to high temperature could help to promote the southward movement of *Paeonia* plants. And *P. suffruticosa* ‘Zhi Hong’ was more high-temperature tolerant, which was attributed to its stable cell membrane, robust antioxidant system, and efficient photosynthetic system [115].

Further comparison of the transcriptomes of *P. lactiflora* ‘Meigui Zi’ (high-temperature-sensitive) and ‘Chi Fen’ (high-temperature-tolerant) suggested that high expression of chlorophyll degradation genes *PlSGR* and *PlSGRL* might be related to high-temperature tolerance [116]. Heat shock proteins (HSPs) play a key role in improving high-temperature tolerance. For *P. suffruticosa*, PsHSP17.8, PsHSP21, and PsHSP27.4 significantly improved high-temperature tolerance by enhancing superoxide dismutase (SOD) activity and proline content [117]. Similarly, PlHSP70 in *P. lactiflora* also demonstrated positive regulation of high-temperature tolerance [118]. In addition, *P. lactiflora* mitogen-activated protein kinase PlMAPK1 played a positive regulatory role in signal transduction in response to high-temperature stress [119]. And PlFabG increased the proportion of FAs, thereby stabilizing cell membranes and helping *P. lactiflora* to resist high-temperature stress [120]. As far as transcription factors were concerned, *P. lactiflora* bHLH transcription factor PlbHLH87 effectively protected the cell membrane and photosystem II from high-temperature injury by reducing ROS accumulation [121]. And *P. lactiflora* AP2/ERF transcription factor PlTOE3 promoted the production of melatonin by binding to the *PlTDC* promoter, which in turn enhanced high-temperature tolerance [122]. Notably, *P. lactiflora* WRKY transcription factor PlWRKY47 formed homo- and heterodimers with PlWRKY72, which directly activated the expression of *PlGAPC2* to improve high-temperature tolerance through inhibiting ROS generation [123]. These studies would provided a basis for breeding high-temperature-tolerant *Paeonia* varieties (Fig. 4B).

#### Other abiotic stress

In addition to drought stress and high-temperature stress, there have been some studies on low-temperature, salt, and waterlogging stresses. Low-temperature tolerance of *P. suffruticosa* significantly depended on anthocyanin and polyunsaturated fatty acid biosynthesis during bud sprouting (Fig. 4C). In detail, PsMYB113 and PsMYB123 directly activated the expression of *PsDFR* and *PsANS* to promote the anthocyanin biosynthesis, while the bZIP transcription factor PsDPBF3 specifically activated the expression of *PsFAD3* to increase the biosynthesis of ALA [124]. *PlWRKY70* sensitively responded to three abiotic stresses, with its expression significantly induced by low-temperature and salt stresses, but inhibited by waterlogging stress [125]. Furthermore, *PlTDC* conferred salt stress tolerance to *P. lactiflora* by promoting melatonin biosynthesis [104].

### Biotic stress

In recent years, with the expansion of pot planting scale in greenhouse, the disease situation of *Paeonia* plants was aggravated in China. Until now, more than 20 kinds of fungal diseases of *Paeonia* plants have been reported. The gray mold caused by *Botrytis cinerea* can result in necrotic leaves, sunken stems, and rotten petals of *Paeonia* plants. In *P. suffruticosa*, plant disease resistance was found to be related to flowering time, with early-flowering cultivars being more resistant to gray mold than late-flowering cultivars [126]. And some genes related to plant–pathogen interaction, secondary metabolic synthesis, and antioxidant system resisting gray mold have been identified in *P. lactiflora*; their expression sharply increased in the resistant cultivar ‘Zi Fengyu’ at early infection but remained relatively low in the susceptible cultivar ‘Da Fugui’ [127].

Leaf spot disease caused by *Alternaria tenuissima* is another fungal disease in *Paeonia* plants, which can lead to leaf scorching and seriously affect photosynthesis. Comparing the defense performance of *P. lactiflora* ‘Zi Fengyu’ (resistant) and ‘Heihai Botao’ (susceptible), the former exhibited faster a hypersensitive response with significantly elevated antioxidant and defense enzyme activities. Furthermore, the expression of multiple pathogenesis-related genes (*PlPR1*, *PlPR2*, *PlPR4B*, *PlPR5*, and *PlPR10*) and *WRKY*s (*PlWRKY13* and *PlWRKY65*) was more strongly induced in ‘Zi Fengyu’ [128]. Previously, virus-induced gene silencing (VIGS) technology demonstrated that *PlWRKY13* and *PlWRKY65* were involved in the JA- and SA-mediated resistance pathways of *P. lactiflora* ‘Da Fugui’ against fungal infections [129, 130]. These studies highlight the crucial regulatory roles of *PlWRKY13* and *PlWRKY65* in resistance to leaf spot disease (Fig. 4D).

In stress resistance research, studies on drought and high-temperature stress are advanced but mostly focus on transcriptional regulation. However, crucial modification pathways like phosphorylation and ubiquitination remain under-explored. Compared to drought stress, the understanding of high-temperature stress regulation is limited, primarily focusing on ROS response. To gain a more comprehensive view, it is essential to integrate multi-omics technologies, such as transcriptomics, proteomics, and metabolomics. Furthermore, abiotic stresses like waterlogging, low-temperature, and salt stresses also significantly affect the growth, development, and industrial progress of *Paeonia* plants, yet research on these stresses lags. Future studies must deepen the exploration of their response mechanisms. Regarding biotic stress, transcriptomic data have initially identified candidate genes associated with resistance to gray mold and leaf spot diseases. Functional validation of their roles in disease resistance networks holds theoretical and applied promise. More systematic research will enrich our understanding of plant stress responses and provide crucial support for stress-tolerant breeding and industrial development of *Paeonia* plants.

## Molecular breeding technology

### Molecular marker

Molecular markers are one kind of genetic indices based on nucleotide sequence variations, which can directly detect individual differences at the DNA level while eliminating environmental effects [131]. With the development of next-generation sequencing technology, more and more molecular markers have been developed, of which the most commonly used in *Paeonia* plants are simple sequence repeat (SSR), sequence-related amplified polymorphism (SRAP), and random amplified polymorphic DNA (RAPD). Currently, these molecular markers have been applied to study cultivar identification, genetic origin, genetic diversity analysis, genetic map construction, and important trait localization in *Paeonia* plants.

With the prolonged artificial cultivation, numerous cultivars of *Paeonia* plants have emerged. Classifying these cultivars scientifically not only aids in the preservation and management of germplasm resources, but also provides a theoretical foundation for their further utilization. For example, two sequence tagged site markers were developed using RAPD markers, including an LuDeB marker for detecting genetic relationships with *Paeonia lutea* and *P. delavayi*, and an HPB marker for identifying cpDNA of *P. lactiflora* in intersubgeneric hybrid cultivars [132]. Zhu et al. developed and validated 20 SSR markers suitable for tree peonies that adapt to growing in the Jiangnan region, thus facilitating research on the genetic diversity, breeding practices, and germplasm innovation of tree peony varieties in this area [133]. Furthermore, the first genetic assessment of the domestication history of *P. ostii* was conducted using SSR markers, revealing that Shaanxi was its main genetic diversity center, and there were multiple independent domestication origins in Shaanxi and Tongling, Anhui province, which were subsequently introduced over long distances to Shandong, Henan, and Hunan provinces [134]. Another SSR marker-based genetic diversity analysis showed that, of nine wild tree peony species, *P. lutea* and *P. delavayi* exhibited high diversity and complexity, while the other seven, notably *Paeonia spontanea* and *P. ludlowii*, faced grave survival threats, needing urgent wild resource conservation [135]. These results would provide important auxiliary breeding tools and theoretical guidance for evaluating the genetic diversity, breeding practices, and germplasm innovation of *Paeonia* plants.

### Genetic map

The genetic map serves as a crucial research tool for non-model species with relatively limited genomic information. It not only helps to analyse the genome structure and precisely locate the loci that influence target traits but also provides key marker information for molecular breeding. Based on the specific-locus amplified fragment sequencing (SLAF-seq), the first high-density genetic map for tree peony was constructed from F1 population of *P. ostii* ‘Fen Dan’ × *P. suffruticosa* ‘Hong Qiao’. The map encompassed five linkage groups with a total length of 920.699 centimorgans (cM), an average intermarker spacing of 0.774 cM, 1115 ‘SNP-only’ markers, 18 ‘InDel-only’ markers, 56 ‘SNP&InDel’ markers, and 37.85% of markers showing significant segregation distortion (*P* < 0.05) [136]. This distortion occurs when the ratio of genotypes observed in segregating populations deviates from the expected Mendelian segregation pattern, greatly affecting the accuracy of genetic mapping. For example, a genetic linkage map was constructed from F1 population of *P. ostii*‘Feng Dan’ × *P. suffruticosa* ‘Xin Riyuejin’ using SSR markers, which contained only 35 valid SSR markers and a total length of 338.2 cM, with a segregation distortion as high as 74.3% [137]. Therefore, it is necessary to construct a higher-density genetic map with large-scale markers to locate and analyze QTLs associated with important traits. When genotyping-by-sequencing (GBS) technology was used, a genetic linkage map constructed also from F1 population of *P. ostii* ‘Feng Dan’ and *P. suffruticosa* ‘Xin Riyuejin’ spanned 13 175.5 cM with an average distance of 3.406 cM between adjacent markers, which greatly increased the marker density of the genetic map and reduced the aberration rate (24.84%) [138]. Additionally, the black or dark purple *P. Suffruticosa* cultivars ‘Qinglong Wo Mochi’ and ‘Mo Zilian’ were selected as parents, and the F1 population was used to construct the high-density genetic maps of the male parent and female parent using restriction site associated DNA sequencing (RADseq), which covered 965.69 and 870.21 cM, with the average marker intervals of 0.66 and 1.10 cM along the seven and five linkage groups, respectively [139].

Although preliminary genetic maps have been constructed for three different parental populations (Table 2), the current study is still insufficient due to the challenges of limited number of offspring, incomplete segregation of phenotypic traits, and few and unevenly distributed of markers. What is more, no complete genetic map for herbaceous peony has been published so far; therefore, the study of genetic mapping for *Paeonia* plants needs to be further deepened and expanded.

**Table 2:** Genetic map information of *Paeonia* plants.

**Code**	**Cross**	**Marker type**	**Marker number**	**Genetic distance (cM)**	**Average distance (cM)**	**Segregation distortion**	**Reference**
1	*P. ostii* ‘Feng Dan’ *× P. suffruticosa* ‘Hong Qiao’	SLAFseq	1189	920.699	0.774	450 (37.85%)	[136]
2	*P. ostii* ‘Feng Dan’ × *P. suffruticosa* ‘Xin Riyuejin’	SSR	35	338.2	9.7	26 (74.3%)	[137]
3	*P. *ostii** ‘Feng Dan’ × *P. suffruticosa* ‘Xin Riyuejin’	GBS	3868	13 175.502	3.406	961 (24.84%)	[138]
4	*P. Suffruticosa* ‘Qinglong Wo Mochi’ × *P. Suffruticosa* ‘Mo Zilian’	RADseq	Female 1471	965.69	0.66	972 (66.08%)	[139]
			male 793	870.21	1.10	107 (13.49%)	

### Localization of QTLs

Based on the constructed genetic map and abundant phenotypic data, molecular markers for some key traits of tree peony have been localized. On the one hand, Guo *et al.* performed QTL analysis based on linkage mapping for 19 phenotypic traits in the F1 offspring and identified 11 QTLs associated with the traits of bud number, leaf length, flower number, pod height, pod diameter, and flower diameter [137]. And Zhang *et al*. detected a total of six QTLs based on the high-density genetic map for four phenotypic traits including number of flowers, petal length, number of petals, and flowering time [138]. On the other hand, based on association mapping, Guo *et al*. investigated 19 quantitative flower and fruit traits using 81 EST-SSR markers; P280, PS2, PS12, PS27, PS118, PS131, and PS145 might be considered potential loci to increase the yield of *P. rockii* [140]. In addition, Peng *et al*. found 86 Genome-Wide Association Studies (GWAS)-related cis-eQTLs associated with the number of petals, stamens, and carpels based on 271 representative *P. suffruticosa* cultivars, and 19 floral organ number-related hub genes with 121 cis-eQTLs were obtained, which helped to understand the evolution of floral organ number in *P. suffruticosa* [141].

At the current stage, genetic mapping on key traits of *Paeonia* plants is still limited, especially for herbaceous peony, which is almost blank. This seriously limits the promotion and application of molecular marker-assisted breeding technology (MAS) in *Paeonia* plants. Therefore, future research is urgently needed to focus on the development of QTLs for key traits, such as flower color, flower fragrance, petal number, stem strength, and seed yield.

### Tissue culture and genetic transformation system

Transgenic technology, an integral part of molecular breeding, serves as an effective method for validating gene function and creating new varieties by precisely integrating exogenous genes into the genome of a targeted organism. Plant tissue culture is the basis for establishing a stable homologous transformation system. Scale buds, cotyledons, petioles, seed embryos, floral organs, leaves, and stem segment are all explant materials for inducing callus of *Paeonia* plants [142–150]. However, serious browning, the difficulties in differentiation and rooting, and low regeneration efficiency in the regeneration system all inhibit the development of transgenic technology of *Paeonia* plants. Attempts have been made to solve some of the problems by means of exogenous pretreatments, adding antibrowning agents, changing the types of mediums and plant growth regulators, and exploring their underlying mechanisms [151]. For example, Zhang *et al*. speculated a potential link between the undifferentiated state of embryogenic callus and hypermethylation, whereas the emergence of rooting plantlets in tissue culture could be correlated with demethylation in *P. ostii* [152]. And *PoWOX*, *PoBBM*, and *PoGPT1* were hypothesized to promote somatic embryogenesis and callus formation of *P. ostii* [153–155]. Unfortunately, even with many explorations, *Paeonia* plants are still making slow progress in establishing mature tissue culture system. At present, only *P. ostii* and ‘High Noon’ have established the preliminary propagation technologies based on tissue culture [156–158].

Furthermore, the development of homologous transgenic system for *Paeonia* plants faces further obstacles. In 2018, Wei used grafting to obtain the first complete *P. ostii* with exogenous genes through *in vitro* regeneration, but the method has not been widely used [5]. As for herbaceous peony, although the *PlIpt* gene was successfully introduced into the callus of *P. lactiflora* ‘Fen Yunu’ by *Agrobacterium*-mediated method in 2007, due to the lack of mature tissue culture system, no research has fully achieved the cultivation of transgenic plants in the following years [159–161].

In the absence of mature homologous stabilization system, gene function was often verified in model plants. But in recent years, homologous transient overexpression technology has also been applied in young tissues of *Paeonia* plants to verify gene function. The seedlings of *P. lactiflora* ‘Fen Yunu’ × ‘Fen Yulou’ with 2- to 3-cm bud length achieved the highest transient transformation efficiency (93.3%) when infected with 1.2 OD_600_  *Agrobacterium* resuspension solution for 12 h, followed by 3 days of coculture in darkness [162]. VIGS technology has also been employed to validate gene function in *Paeonia* plants. In 2019, Xie *et al.* pioneered its use in *P. ostii*, discovering that vacuum infiltration more effectively penetrated *Agrobacterium* than syringe infiltration [163]. Since then, this technique has been widely used for gene silencing studies in buds, petals, leaves, and even whole *Paeonia* plants.

In summary, preliminary progress has been achieved on molecular breeding technology for *Paeonia* plants, including the development of molecular markers, the construction of genetic maps, and the localization of QTLs for tree peony. These advancements provide a crucial foundation for genetic dissection of important traits and MAS. However, research in these areas for herbaceous peony remains almost vacant, requiring more investment to achieve synergistic development between tree and herbaceous peony. By increasing marker density and optimizing progeny population design, a more precise genetic map can be constructed, providing more comprehensive support for QTL localization of important traits such as flower color, fragrance, stem strength, and seed yield. On this basis, combined with stable genetic transformation technology, there is potential to truly realize transgenic breeding and cultivate new varieties with superior traits. However, the current state of tissue culture and gene transformation systems for *Paeonia* plants is relatively immature, severely hindering the progress of transgenic breeding. There is an urgent need to explore and develop new methods to address these challenges.

## Perspectives

Currently, the production of *Paeonia* plants heavily relies on traditional breeding, which makes it urgent to develop modern molecular breeding technology to meet growing market demands. The genome of *Paeonia* plants could provide the basis for genetic studies. In 2020, the publication of the first genome draft of *P. suffruticosa* announced the genomic era for genetic studies [10]. Subsequently, higher-quality genomes of two tree peony species, *P. ostii* and *P. ludlowii*, were completed, which provided new insights into environmental adaptation and species evolution [11, 13]. Alternatively to de novo genome assembly, whole-genome sequencing could identify genetic variations such as SNPs at low cost, exemplified by its revelation of the parental contribution in *Paeonia* Itoh hybrids [12]. Compared with herbaceous peony, whose whole genome sequencing has yet to be initiated, the genome research of tree peony has advanced significantly, but there is still room for improvement in sequencing accuracy and data application. By integrating third-generation sequencing technologies (such as PacBio and Nanopore) with advanced methods like pangenome analysis and Hi-C, we can effectively address the genome sequencing challenges that arise from high ploidy, homologous polyploidy, and the vast genome size of *Paeonia* plants [9]. Furthermore, the use of genomic data to construct high-density linkage maps and perform GWAS analysis can identify key genes affecting specific phenotypic traits, which in turn can effectively promote molecular breeding.

Significant progress has been made in flower color formation of *Paeonia* plants. However, research on other regulatory mechanisms, particularly those related to biotic and abiotic stress responses, is lagging, necessitating further elucidation of the relevant genetic regulatory pathways and mechanisms. At present, the regulatory studies of functional genes mainly focus on the transcriptional level. For the more complex epigenetic mechanisms, only initial exploration has been performed, such as the effect of DNA methylation elimination in petal blotch formation [79] as well as the impact of ubiquitin degradation on bud dormancy [21, 22] or chalcone biosynthesis [63]. However, the specific functional mechanisms of other epigenetic modifications in *Paeonia* plants, such as the crucial role of phosphorylation in signal transduction and the potential influence of acetylation on the activity of metabolic enzymes, still remain unexplored [164, 165]. In addition, most of the existing omics studies have been limited to the transcriptome, with only a few studies fully integrating two or more omics approaches. Future integration of multi-omics data with phenotypic information could help screen genes or pathways associated with key traits and facilitate targeted genetic modifications.

Due to the absence of genetic transformation system in *Paeonia* plants, gene function validation mainly relies on *Arabidopsis* and tobacco. Establishing such a system is vital for gene function analysis, germplasm resource development, and molecular breeding. Future study should focus on understanding physiological and biochemical characteristics, optimizing tissue culture conditions, and improving gene transformation strategies of *Paeonia* plants. Exploring magnetic nanoparticle-mediated transgenic technology [166, 167] and efficient *Agrobacterium tumefaciens*-mediated methods, like cut-dip-budding delivery system [168] and the regenerative activity-dependent *in planta* injection delivery (RAPID) method [169], could skip the barriers of difficult tissue culture, potentially leading to a stable genetic transformation system. Additionally, more efforts should be made to apply molecular markers, develop genetic maps and localization of QTLs, and promote synergistic development between tree peony and herbaceous peony. In the future, the integration of multiple breeding techniques including MAS breeding, transgenic breeding, and traditional breeding would enable new *Paeonia* varieties to be tailored to market demands.

## Supplementary Material

Web_Material_uhaf090

## Data Availability

No new data was used for the research described in the article.
