# Supplementary material for: Unlocking the molecular secrets of Paeonia plants: advances in key gene mining and molecular breeding technology
Source: Hortic Res. 2025 Apr 30;12(7):uhaf090. doi: 10.1093/hr/uhaf090 (PMC12064954; doi:10.1093/hr/uhaf090)

Supplemental figure for review 1 Sequence comparison of key flower color regulatory genes in tree peony and herbaceous peony.


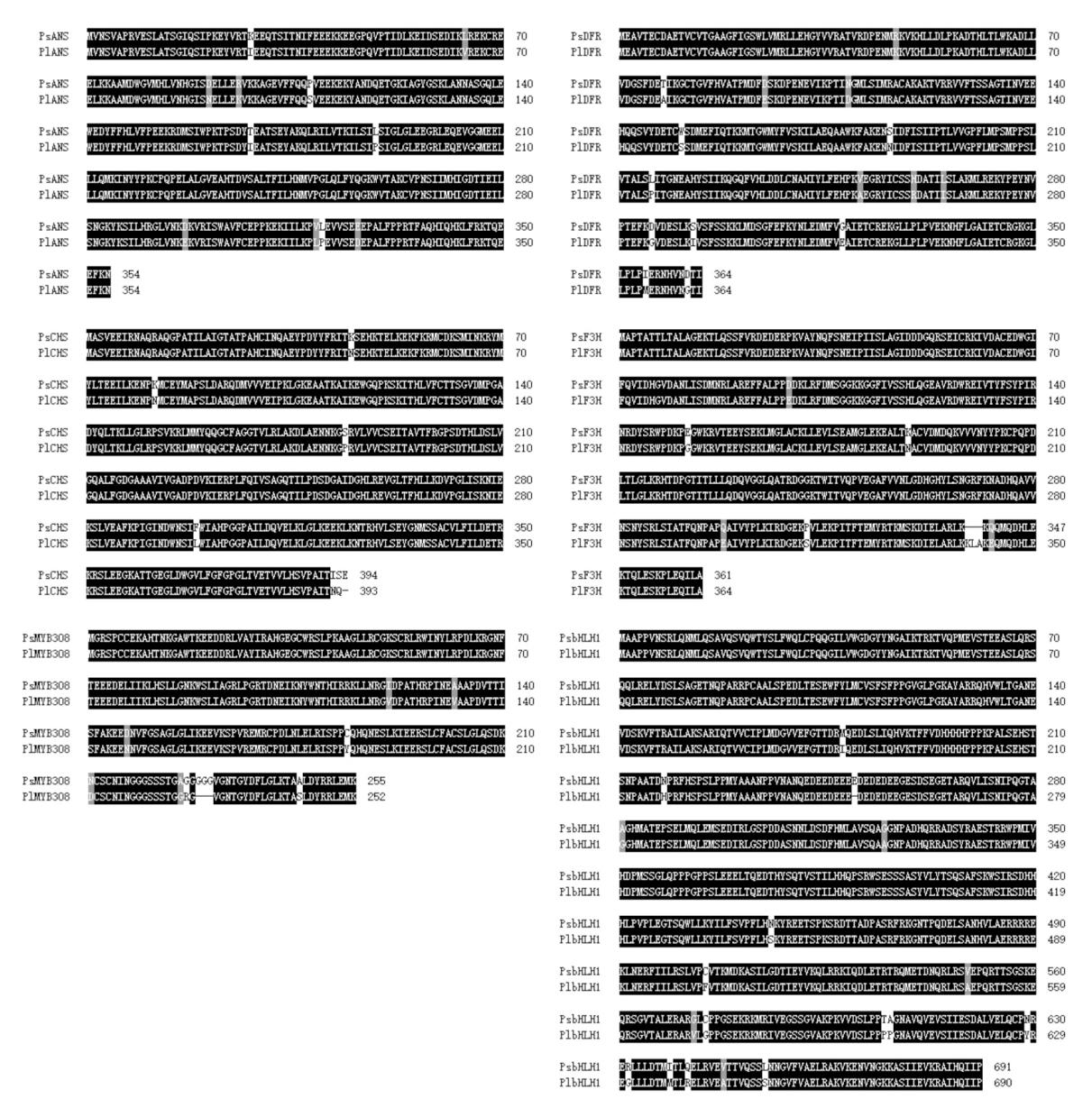

Supplement: Web_Material_uhaf090 [file web_material_uhaf090.zip › Supplemental figure for review 1.docx]
