# Supplementary material for: Unlocking the molecular secrets of Paeonia plants: advances in key gene mining and molecular breeding technology
Source: Hortic Res. 2025 Apr 30;12(7):uhaf090. doi: 10.1093/hr/uhaf090 (PMC12064954; doi:10.1093/hr/uhaf090)
Supplement: Web_Material_uhaf090 [file web_material_uhaf090.zip › Supplementary Table 1.docx]

**Supplementary Table 1** Full name of the genes.

| **Abbreviation** | **Full name** |
| --- | --- |
| *AACT* | Acetyl-CoA C-acetyltransferase |
| *ABF* | ABA-responsive element binding factors |
| *ABI* | ABA-insensitive |
| *ACL* | ATP-citrate lyase |
| *ACO* | 1-aminocyclopropane-1-carboxylate oxidase |
| *ADH* | Alcohol dehydrogenase |
| *AG* | Agamous |
| *ANS* | Anthocyanidin synthase |
| *AP* | Apetala |
| *ASIL* | *Arabidopsis* 6b-interacting protein-like |
| *ATL* | *Arabidopsis* toxicos en levadura |
| *BBM* | Baby boom |
| *bHLH* | Basic helix-loop-helix |
| *bZIP* | Basic leucine zipper |
| *C/EBPα* | CCAAT/Enhancer-binding protein alpha |
| *C4H* | Cinnamate 4-hydroxylase |
| *CAB* | Chlorophyll a/b-binding |
| *CCoAOMT* | Caffeoyl-CoA-*O* methyltransferase |
| *CHI* | Chalcone isomerase |
| *CHS* | Chalcone synthase |
| *CO* | Constans |
| *COMT* | Caffeic acid *O*-methyltransferase |
| *Cu/Zn-SOD* | Cu/Zn-Superoxide dismutase |
| *CYCD* | D-type cyclins |
| *DFR* | Dihydroflavonol 4-reductase |
| *DGAT* | Diacylglycerol acyltransferase |
| *DPBF3* | bZIP transcription factor |
| *DREB* | Dehydration responsive element binding protein |
| *DXR* | 1-deoxy-D-xylulose-5-phosphate reductoisomerase |
| *DXS* | 1-deoxy-D-xylulose-5-phosphate synthase |
| *EBB* | Early budbreak |
| *EP* | Epithelial-specific secreted glycoprotein |
| *F3’H* | flavanone 3’-hydroxylase |
| *F3H* | Flavanone 3-hydroxylase |
| *FabG* | 3-oxoacyl-ACP reductase |
| *FAD* | Fatty acid desaturase |
| *FBA* | Fructose 1,6-bisphosptase |
| *F-box* | F-box family gene |
| *FD* | Basic region leucine zipper member |
| *FFL* | F-box/FBD/LRR-repeat |
| *FLC* | Flowering locus C |
| *FLS* | Flavonol synthase |
| *FT* | Flowering locus T |
| *FUS* | Fusca |
| *GAPC* | Cytosolic glyceraldehyde‐3‐phosphate dehydrogenase |
| *GGPS* | Geranylgeranyl diphosphate synthase |
| *GPT* | Glutamic pyruvic transaminase |
| *GSTF* | Anthocyanin-related glutathione S-transferase |
| *HCT* | Hydroxycinnamoyl transferase |
| *HDR* | 4-hydroxy-3-methylbut-2-enyl diphosphate reductase |
| *HDS* | 4-hydroxy-3-methylbut-2-enyl diphosphate synthase |
| *HMGR* | 3-hydroxy-3-methyl glutaryl coenzyme A reductase |
| *HSP* | Heat shock proteins |
| *Ipt* | Isopentenyl transferases |
| *LAC* | Laccase |
| *LACS* | Long-chain acyl-CoA synthetases |
| *LEC* | Leafy cotyledon |
| *LIS* | Linalool synthetase |
| *LPAAT* | Lysophosphatidic acid acyltransferase |
| *MAPK* | Mitogen-activated protein kinase |
| *MYB* | R2R3-MYB family transcription factor |
| *MYS* | 1 myrcene synthase |
| *NCED* | 9-cis epoxycarotenoid dioxygenase |
| *NF-YC2* | Nuclear factor Y |
| *OLE* | Oleosin |
| *P5CS* | Pyrroline-5-carboxylate synthase |
| *PDAT* | Phospholipids: diacylglycerol acyltransferase |
| *PIN* | Pin-formed |
| *PM* | Plasma membrane |
| *PMK* | Phosphomevalonate kinase |
| *PR* | Pathogenesis related protein |
| *RGL* | RGA-like |
| *RING-H2* | RING-H2 finger protein |
| *SGR* | Stay-green rice |
| *SLR* | Slender rice like |
| *SOC* | Suppresspor of overexpenssion of constans |
| *SPL* | Squamosa promoter binding protein-like |
| *TDC* | Tryptophan decarboxylase |
| *TFL* | Terminal flower |
| *TOE* | Target of early activation tagged |
| *TPS* | Terpene synthase |
| *UF3GT* | Flavonoid 3-*O*-glycosyltransferase |
| *VIN* | Vernalization insensitive |
| *VQ* | Valine-glutamine (VQ) motif-containing protein |
| *WD* | WD-repeat protein |
| *WOX* | WUSCHEL-related homeobox |
| *WRI* | Wrinkled |
| *WRKY* | WRKY family transcription factor |
| *XTH* | Xyloglucan endotransglucosylase/hydrolase |
| *ZFP* | Zinc-finger protein |
